# Excision of Intra-articular Knee Heterotopic Ossification Using a 70° Arthroscope

**DOI:** 10.1155/2024/9998388

**Published:** 2024-06-06

**Authors:** Alexander J. Hoffer, Eugenia A. Lin, Maziyar A. Kalani, Mark K. Lyons, Meghan Richardson

**Affiliations:** Department of Orthopedic Surgery Mayo Clinic, 5881 E. Mayo Blvd., Phoenix, AZ 85054, USA

## Abstract

Heterotopic ossification is ectopic lamellar bone formation within soft tissue and can result in significant functional limitations. There are multiple underlying etiologies of HO including musculoskeletal trauma and traumatic brain injury. Intra-articular HO of the knee is rare and is typically located within the cruciate ligaments. We report a case of a 24-year-old female who presented with worsening right knee pain and limited knee extension two and a half years after a motor vehicle crash with multiple lower extremity fractures. Physical examination of the knee revealed anterior pain, limited extension, and a palpable infrapatellar prominence. Imaging showed a retropatellar tendon, intra-articular excrescence of bone proximal to the anterior tibial plateau. Diagnostic arthroscopy with a 70° arthroscope identified HO at the proximal anterior tibial plateau, which was excised with a high-speed burr under direct visualization. At the three-month follow-up, the patient remained asymptomatic and returned to sport. Retropatellar tendon, intra-articular anterior knee HO is a rare but debilitating clinical entity that can be successfully and safely managed with excision under direct visualization using a 70° arthroscope.

## 1. Introduction

Heterotopic ossification (HO) is ectopic lamellar bone formation in soft tissue that can range from functionally insignificant to highly dysfunctional. Etiologies of HO include trauma, central nervous system injury such as cerebrovascular accident or traumatic brain injury, and thermal injury [1]. The pathogenesis of HO is largely unknown but likely involves the conversion of progenitor cells to bone forming cells due to cellular responses to changes in the local tissue environment [1]. Patients with post-traumatic HO may present with restricted range of motion (ROM), nerve entrapment, skin ulceration, and persistent pain and often require HO surgical excision [2].

Knee arthroscopy is a common minimally invasive surgical procedure to manage intra-articular pathology. A 30° arthroscope is used for most procedures, but a 70° arthroscope may be used occasionally to facilitate visualization around structures such as when identifying the tibial footprint during posterior cruciate ligament (PCL) reconstruction [3]. The applications of a 70° arthroscope in knee arthroscopy continue to increase as technology, and our understanding of knee pathologies continues to advance.

We present a rare case of post-traumatic, retropatellar tendon, intra-articular, knee HO and a safe method of surgical excision under direct visualization using a 70° arthroscope.

## 2. Case Presentation

A 24-year-old female presented for evaluation of right knee pain, crepitus, and limited extension. The patient's past medical history was significant for a motor vehicle crash two and a half years prior to presentation during which she fractured her right ankle, right tibia and fibula, right femoral shaft (open) and femoral neck, right metacarpals two through five, left femur, left forearm, and multiple level spine fractures including C2, C4 through C7, L4, and her coccyx. Additionally, the patient sustained a mild traumatic brain injury. She was admitted for three months at an outside hospital after her initial injury. During her hospitalization, she underwent stabilization of her right femur and tibia with intramedullary (IM) nails, right femoral neck operative reduction and internal fixation, right ankle open reduction and internal fixation, left femur IM nail, left radius and ulna open reduction and internal fixation, and percutaneous fixation of her second through fifth right metacarpal fractures. She underwent subsequent manipulation under anesthesia of the right knee without arthroscopic debridement eight weeks after her initial surgeries to improve her knee range of motion. She was discharged home and underwent progressive physical and occupational therapy for six months after her initial injury.

The patient presented to outpatient orthopedic surgery and sports medicine clinic two and a half years after her initial injury with a chief complaint of right knee pain and restricted extension. She experienced right-sided crepitus during knee flexion for one year and worsening anterior knee pain for three months. The pain occurred primarily when transitioning from a sitting to standing position. Additionally, the patient stated that she did not regain full passive or active knee extension since her initial hospitalization. She denied any mechanical symptoms or instability.

On general examination, she appeared an overall healthy female of stated age. Local examination of the right knee revealed a fully healed longitudinal suprapatellar incision with no effusion or signs of infection. There was tenderness to palpation of an anterior, infrapatellar prominence, and restricted range of motion from 0° of extension to 130° of flexion compared to 5° of hyperextension of the contralateral knee. She had a nontender medial and lateral joint line, a stable ligamentous examination, and a normal distal neurovascular examination.

**Figure 1:**
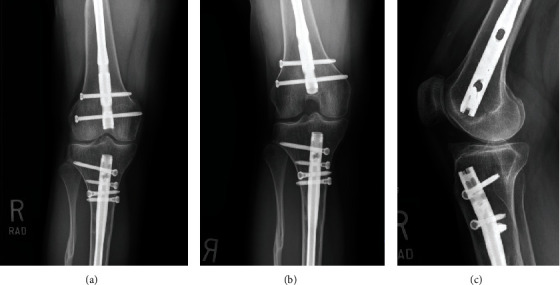
Preoperative right knee radiographs demonstrate retrograde femoral rod fixation and antegrade tibial rod fixation: (a) anterior-posterior view; (b) posterior-anterior flexion view; (c) lateral view, which best demonstrates the anterior intra-articular heterotopic.

Plain film radiographs of the right knee obtained two and a half years after initial injury demonstrated prior right retrograde femoral IM nail, right antegrade tibial IM nail, and a retropatellar tendon, 15 × 24 mm bony prominence along the anterior tibial plateau suggestive of heterotopic ossification. No acute fractures or dislocations were evident. The prior right tibial shaft and proximal fibular shaft fractures were well-healed. There was no evidence of a peri-implant complication (Figure 1).

**Figure 2:**
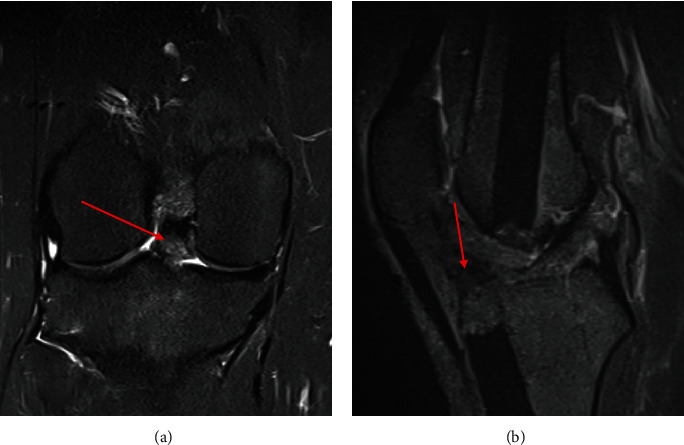
Preoperative right knee magnetic resonance imaging study. The red arrows indicate the anterior intra-articular heterotopic ossification: (a) select coronal T2 image; (b) select sagittal T2 image. A red arrow indicates the anterior intra-articular heterotopic ossification in both images.

Metal reduction magnetic resonance imaging (MRI) of the right knee revealed an osseous excrescence (17 × 8 × 14 mm) on the anterior tibial plateau and an area of fibrosis (12 × 11 × 7 mm) within Hoffa's fat, consistent with arthrofibrosis. MRI also redemonstrated femoral and tibial IM nails and full-thickness chondral loss on the posterior lateral tibial plateau (Figure 2). A bone scan was not obtained.

Nonoperative and operative treatments were discussed with the patient. Nonoperative options included oral nonsteroidal anti-inflammatory medications (NSAIDs) and a right knee intra-articular corticosteroid injection. The patient declined NSAIDs due to a previous adverse reaction from them. The surgical options included diagnostic arthroscopy, fat pad and arthrofibrosis debridement, and excision of heterotopic ossification. The patient chose to proceed with surgical intervention after the risks and alternatives for all treatment options were explored thoroughly.

**Figure 3:**
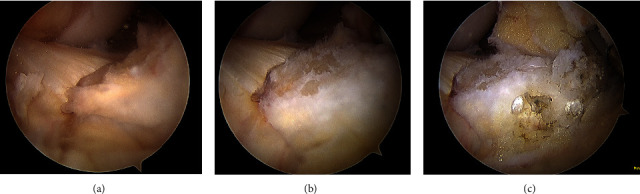
Intra-operative views using a 70° arthroscope of the anterior intra-articular heterotopic ossification: (a) before debridement of fat pad and dissection away from patellar tendon; (b) after partial fat pad debridement; (c) after complete fat pad debridement and dissection away from patellar tendon.

The patient underwent a right knee diagnostic arthroscopy. A large area of retropatellar tendon HO extending posteriorly to the border of the anterior cruciate ligament was visualized (Figure 3).

**Figure 4:**
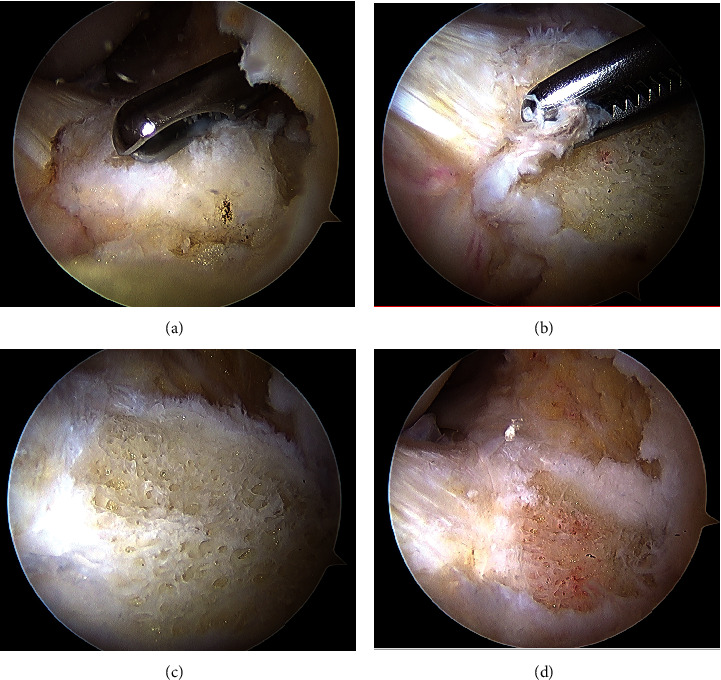
Intraoperative views using a 70° arthroscope of the anterior intra-articular heterotopic ossification: (a) after fat pad debridement and dissection away from the patellar tendon before excision with burr; (b) after removal of surrounding periosteum and synovium with shaver; (c) after complete HO resection with a burr; (d) global view after complete HO resection.

Careful dissection of the fat pad and patellar tendon off this bony prominence with combined blunt dissection and radiofrequency ablation was completed under direct visualization through a 70° arthroscope. The anterior HO was excised with a 4.5 mm arthroscopic hooded burr under direct visualization with a 70° arthroscope. Throughout excision, the hood of the burr was positioned against the patellar tendon to avoid damaging it (Figure 4).

**Figure 5:**
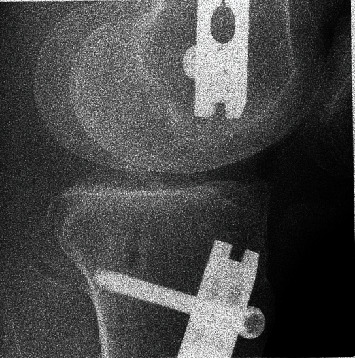
Intraoperative fluoroscopic lateral image of the right knee to confirm adequate resection of the anterior intra-articular heterotopic ossification.

**Figure 6:**
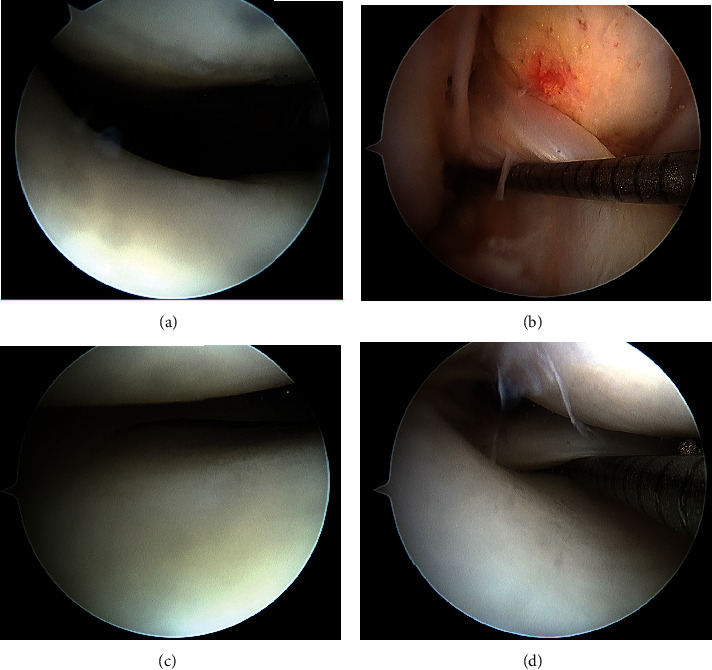
Diagnostic arthroscopy after heterotopic ossification resection to confirm the absence of loose bodies: (a) patellofemoral joint; (b) notch, anterior cruciate ligament right of the probe; (c) medial compartment with medial femoral condyle, medial meniscus, and medial tibial plateau visualized; (d) lateral compartment with lateral femoral condyle, lateral meniscus, with the probe under the meniscal root, and lateral tibial plateau visualized.

Intraoperative fluoroscopy was utilized to confirm complete HO resection (Figure 5). Subsequent ROM examination under anesthesia revealed no block to extension and a ROM of 5° of hyperextension to 130° of flexion, symmetric to the contralateral side. No loose bodies were noted during postresection diagnostic arthroscopy (Figure 6). Subcutaneous analgesic was injected around the portal sites after they were closed in standard fashion. The patient was placed in a compressive bandage. There were no immediate postoperative complications.

**Figure 7:**
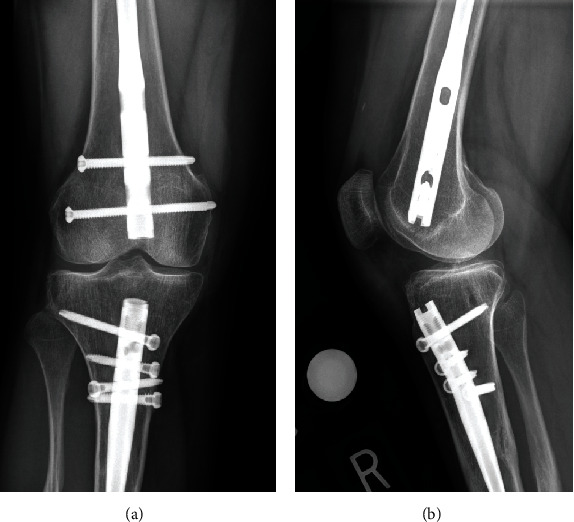
Three-month postoperative radiographs confirming complete resection of the anterior intra-articular heterotopic ossification: (a) anterior-posterior image; (b) lateral image.

No weight bearing or range of motion restrictions was placed after surgery. Progressive physical therapy with ROM exercises, gait rehabilitation, and resistance training was prescribed. Deep vein thrombosis prophylaxis with aspirin 81 mg daily and HO recurrence prophylaxis with indomethacin 25 mg daily were prescribed. At her two-week follow-up visit, the patient's incision was healed, pain was improved, she ambulated with crutches, and she continued physical therapy. At her three-month follow-up visit, the patient's postoperative pain had resolved, and she was asymptomatic. She returned to amateur level CrossFit competitions without any further discomfort or knee pain. Radiographs of the right knee demonstrated her intact femoral and tibial nails with no evidence of HO recurrence (Figure 7).

## 3. Discussion

We presented a rare case of symptomatic, ROM-restricting, retropatellar tendon, intra-articular knee HO, and a novel application of the 70° arthroscope for HO excision under direct visualization. During this case, careful dissection of arthrofibrotic adhesions between the patellar tendon and HO was necessary to mobilize the tendon and completely excise the HO while keeping the tendon in continuity. This would have been exceedingly difficult using a conventional 30° arthroscope and standard anteromedial and anterolateral arthroscopic portals for visualization. The 70° arthroscope was integral for safe dissection under direct visualization and successful correction of this pathology.

HO is a common complication of orthopedic trauma, especially when there is a concomitant traumatic brain or spinal cord injury [4]. In the initial stages of formation, it may be difficult to diagnose HO because of its nonspecific presentation and radiographic findings [5]. Serum alkaline phosphatase (ALP) may be a useful screening tool in the early stages because it is typically elevated between two and 18 weeks after HO onset [6]. Similarly, three-phase bone scintigraphy is the most sensitive imaging modality to detect immature HO [5]. However, in the context of a chronic, stable, intra-articular lesion with a 2.5-year post-traumatic history, the utility of acute-phase diagnostic modalities is limited. Further, once mature lamellar HO forms, MRI is the modality of choice and can be considered diagnostic if it shows cancellous fat that is hyperintense on T1- and T2-weighted images [7, 8]. Prophylaxis with NSAIDs is widespread practice despite multiple randomized controlled trials that showed little to no improvement in HO formation compared to placebo or radiation [9–11]. A single dose of radiation may provide some benefit, but it is challenging to administer within the 72-hour window from the initial inciting event. From a surgical perspective, a patient with ongoing functional limitation that has exhausted nonoperative management is a candidate for surgical excision; however, surgical timing remains controversial. The excision of a lesion before 6 month after initial onset or while serum ALP levels are still elevated is associated with a higher recurrence risk [12]. On the other hand, excessive delay may be associated with irreversible loss of motion [12]. In our case, the patient presented two and a half years after onset with mature lamellar HO. Given the duration since onset and mature HO structure diagnosed on MRI, there was no need for further diagnostic studies such as a bone scan to evaluate the phase of HO maturation or for postoperative histopathological confirmation of HO [12, 13]. While it is impractical to screen for HO recurrence with routine postoperative serum ALP, it could be considered if a patient was to experience symptom recurrence after surgical excision.

Few cases of noninstrumented native knee intra-articular HO have been reported. The most common presentation involves ossification within the substance of a cruciate or collateral ligament, with or without precipitating trauma [14–19]. However, intra-articular knee HO after a tibial IM nail may be more common than previously believed. A recent retrospective review suggested that postoperative HO is present in 15% of cases after tibial IM nails [20]. Interestingly, despite the high prevalence in this study, no patient had symptomatic HO requiring further surgery. HO after tibial IM nail is commonly associated with a head injury [21–23]. Four case reports have also described intra-articular HO of the knee after retrograde femoral IM nail stabilization [24–27]. In one case, the intra-articular HO was debrided arthroscopically, but the 70° arthroscope was not utilized. This case was also the first to our knowledge to document intra-articular knee HO after combined retrograde femoral nail and antegrade tibial nail on the ipsilateral extremity in the context of a head injury. Since knee HO has been reported after both procedures in isolation, the combined procedure may be at higher risk for postoperative HO, a complication that may be under-reported in the literature.

The indications for a 70° arthroscope continue to increase during knee arthroscopy. Historically, the 70° arthroscope was utilized to identify the tibial footprint and create the tibial tunnel during PCL reconstruction [2]. However, more contemporary indications described include repair of meniscal ramp lesions and synovectomy and debridement of the posterior compartment of the knee [28]. Posterior compartment knee arthroscopy with a 70° arthroscope reversed the treatment algorithm for telangiectatic giant cell tenosynovitis (TGCT). Historically, anterior compartment TCGT was debrided arthroscopically, and posterior compartment TCGT was debrided through open surgery. However, the algorithm shifted to open surgery anterior and arthroscopy posteriorly, or completely arthroscopic management in some cases as joint preservation surgeons improved using a 70° arthroscope posteriorly [29–31]. Similar advances occurred with the treatment of other intra-articular pathology, such as hemophilia-induced synovitis and synovial osteochondromatosis. A 70° arthroscopic technique has also been described with or without the use of a superolateral portal to provide superior visualization of the infrapatellar region and undersurface of the patellar tendon when treating patellar tendinopathy arthroscopically [32]. Similar to the technique described to treat patellar tendinopathy, this case required the use of a 70° arthroscope in the anterior compartment of the knee, which is rare due to easier access to the front of the knee and less surgically correctable pathology anterior relative to the other compartments of the knee.

## 4. Conclusion

Retropatellar, intra-articular knee HO is a rare condition that may occur after ipsilateral concomitant retrograde femoral nail and antegrade tibial nail. Surgical intervention with a 70° arthroscope provides a safe and effective option to excise intra-articular knee HO under direct visualization while protecting the extensor mechanism to restore patient function.

## Data Availability

There is no further relevant data outside what was described in this case report. The identity of the subject of this case report will remain anonymous to observe patient confidentiality.
